# Photobiomodulation of oral fibroblasts stimulated with periodontal pathogens

**DOI:** 10.1007/s10103-021-03331-z

**Published:** 2021-05-15

**Authors:** H. J. Serrage, P. R. Cooper, W. M. Palin, P. Horstman, M. Hadis, M. R. Milward

**Affiliations:** 1 Oral Microbiology Unit, Department of Oral and Dental Science, University of Bristol, Bristol BS1 2LY, UK; 2 School of Dentistry, University of Birmingham, Birmingham, UK; 3 Faculty of Dentistry, Department of Oral Biology, Sir John Walsh Research Institute University of Otago, Dunedin, New Zealand; 4 Philips Research, Eindhoven, Netherlands

**Keywords:** Photobiomodulation, Periodontitis, Fibroblast, Mitochondria, PBM

## Abstract

Photobiomodulation (PBM) utilises light energy to treat oral disease, periodontitis. However, there remains inconsistency in the reporting of treatment parameters and a lack of knowledge as to how PBM elicits its molecular effects in vitro. Therefore, this study aimed to establish the potential immunomodulatory effects of blue and near infra-red light irradiation on gingival fibroblasts (GFs), a key cell involved in the pathogenesis of periodontitis. GFs were seeded in 96-well plates in media + / − *Escherichia coli* lipopolysaccharide (LPS 1 μg/ml), or heat-killed *Fusobacterium nucleatum* (*F. nucleatum,* 100:1MOI) or *Porphyromonas gingivalis* (*P. gingivalis,* 500:1MOI). Cultures were incubated overnight and subsequently irradiated using a bespoke radiometrically calibrated LED array (400–830 nm, irradiance: 24 mW/cm^2^ dose: 5.76 J/cm^2^). Effects of PBM on mitochondrial activity (3-(4,5-dimethylthiazol-2-yl)-2,5-diphenyltetrazolium bromide (MTT) and adenosine triphosphate (ATP) assays, total reactive oxygen species production (ROS assay) and pro-inflammatory/cytokine response (interleukin-8 (IL-8) and tumour growth factor-β1 (TGFβ1)) were assessed 24 h post-irradiation. Data were analysed using one-way ANOVA followed by the Tukey test. Irradiation of untreated (no inflammatory stimulus) cultures at 400 nm induced 15%, 27% and 13% increases in MTT, ROS and IL-8 levels, respectively (*p* < 0.05). Exposure with 450 nm light following application of *P. gingivalis, F. nucleatum* or LPS induced significant decreases in TGFβ1 secretion relative to their bacterially stimulated controls (*p* < 0.001). Following stimulation with *P. gingivalis*, 400 nm irradiation induced 14% increases in MTT, respectively, relative to bacteria-stimulated controls (*p* < 0.05). These findings could identify important irradiation parameters to enable management of the hyper-inflammatory response characteristic of periodontitis.

**Supplementary Information:**

The online version contains supplementary material available at 10.1007/s10103-021-03331-z.

## Introduction

Photobiomodulation (PBM) is defined as the application of light at 400–1100 nm to promote tissue healing and reduce inflammation [1]. One area that has gathered considerable interest in recent years is the application of PBM in the management of periodontitis [2]. Periodontitis is a highly prevalent chronic oral inflammatory disease characterised by the loss of structures surrounding and supporting the tooth. Periodontitis affects 43% of the UK adult population and costs the National Health Service (NHS) an estimated £2.8 billion per annum for patient treatment [3]. Periodontitis not only leads to tooth loss but has also been shown to adversely affect masticatory function, quality of life [4] and systemic health [5]. Furthermore, periodontitis and periodontitis-associated pathogens have been associated with the onset of systemic diseases including infective endocarditis [6], Alzheimer’s disease [7] and colorectal cancer [8]. New treatments are thus urgently required to manage and treat this prevalent disease.

The key aetiological agent in periodontal disease is the oral biofilm. Oral biofilm formation is characterised by the initial adhesion of primary colonisers to the salivary pellicle. This facilitates coaggregation with bridging species including *Fusobacterium nucleatum* (*F. nucleatum*), enabling the colonisation by periopathogenic bacterial species including *Porphyromonas gingivalis (P. gingivalis)* [9, 10]. Rendering, *F. nucleatum* and *P. gingivalis* key species in oral disease associated biofilm formation. In turn, bacterial stimuli can be recognised by receptors localised on the surface of oral cell types including gingival fibroblasts (GFs) culminating in increased cytokine release, leading to the propagation of inflammation associated with periodontitis [11, 12].

GFs are a fundamental cell type in the study of oral disease due to their abundance and their ability to modulate oral inflammation [13]. Healthy GFs also exhibit different responses to stimuli when compared with inflamed GFs, making them a model cell type for elucidating the biomodulatory effects of light [14].

GFs are commonly used to explore the effects of PBM as a model for periodontitis. In particular, near infra-red (NIR) light has been observed to promote cell proliferation [15–17] and decrease markers for inflammation [18–20] from GFs. Comparatively, the effects of blue light have only been reported by relatively few authors, where high doses of blue light (> 100 J/cm^2^) induce increased oxidative stress [21, 22]. However, blue light application (405 nm) was also proven effective as red light (635 nm) in promoting gingival fibroblast cell proliferation at 25 J/cm^2^ [23]. Indicating the possible modulatory capacity of low-dose blue light. The majority of studies evaluate the effects of PBM on GFs following application of a pro-inflammatory stimulus, most commonly lipopolysaccharide (LPS) [18, 19, 24–26]. LPS is a virulence factor located on the surface of gram-negative pathogens including *F. nucleatum* and *P. gingivalis.* However, as species associated with periodontal disease produce a range of virulence factors [27], the biomodulatory effects of PBM in managing inflammation induced by oral biofilm relevant species remain to be elucidated.

The effects of PBM on oral fibroblasts have also been reviewed by Ren et al. [28] and concluded there remain several key issues yet to be addressed. Notably poor experimental design where parameters including wavelength (nm) were incorrectly measured or not reported at all [29], rendering it difficult to draw conclusions regarding the possible benefits of PBM in periodontitis management.

The cellular effects of PBM are also yet to be fully elucidated. Light is proposed to modulate mitochondrial activity via the excitation of complexes of the mitochondrially located electron transport chain (ETC), inducing the production of reactive oxygen species (ROS) and the cells’ energy source, adenosine triphosphate (ATP) as a by-product of the progression of the ETC. In turn, ROS induces the activity of downstream targets including nuclear factor kappa-light-chain-enhancer of activated B cells (NFκB) which modulates the transcription of chemokines, cytokines and growth factors [30]. The levels of activation of NFκB have been shown to correlate with interleukin 8 (IL-8) [31]. IL-8 is a chemokine and periodontitis biomarker, produced by GFs. At high concentrations, IL-8 is instrumental in the recruitment of inflammatory cell types involved in periodontal tissue destruction [32, 33]. But, at low levels, it is reported to modulate cellular homeostasis [34]. Indeed, a number of authors have established the biomodulatory effects of NIR light on IL-8 secretion from GFs. Indeed, NIR light exposure resulted in significant decreases in IL-8 secretion from pro-inflammatory stimulus–treated GFs [18, 19, 25, 35–38]. However, as to whether this effect is linked to changes in mitochondrial activity is yet to be elucidated.

Transforming growth factor-β (TGFβ) signalling is also proposed to be modulated by changes in ROS production. TGFβ is secreted in its inactive state associated with latency-associated peptide (LAP). The dissociation of which is proposed to be induced by increases in ROS (caused by PBM), to form active TGFβ, of which there are three mammalian isoforms where TGFβ1 has been identified as a periodontitis biomarker [39]. PBM has been identified to reduce TGFβ1 in gingival crevicular fluid (GCF, an inflammatory exudate derived from periodontal tissues) following non-surgical periodontal treatment [40]. In turn, TGFβ binds its cell surface receptor TGFβRIII, which then transfers TGFβ to TGFβRI and TGFβRII leading to downstream effects including angiogenesis and fibrosis. However, light-induced changes in TGFβ signalling have only been reported by a handful of authors and are yet to be explored in GFs [30].

Using an array we have previously developed and characterised [41], the aim of this study was to further explore the effects of low-dose PBM at wavelengths spanning the visible to NIR spectrum on mitochondrial activity (ROS and ATP assay) and ROS-induced downstream changes on IL-8 and TGFβ signalling (TGFβ1 and TGFβR1). Furthermore, the effects of PBM on GFs were explored under health- and disease-relevant conditions by exposure to periodontopathogens. Our findings aim to not only improve the understanding of the cellular response of GFs to PBM but also identify irradiation parameters for translation to the clinical management of periodontitis. Where, we provide novel evidence of the modulatory effects of low-dose blue light on gingival fibroblasts and its potential efficacy in the management of oral disease.

## Materials and methods

### LED array design

The LED array was designed using CADsoft EAGLE software, in which 60 centrally located 5 mm epoxy-encased LEDs (Roithner Laserthek, Vienna, Austria) were located within a 96-microwell plate. The array contained ten channels, each emitting different peak wavelengths (400–830 nm, n = 6) and a standardised irradiance value of 24 mW/cm^2^. During irradiation, columns 5 and 7 were switched off to act as non-irradiated controls (depicted in Supplementary Fig. 1). Variable resistors were attached to each wavelength channel to enable independent voltage control to ensure uniform irradiance delivery across the array. To ensure concentric alignment of the array with cell cultures and prevention of potential bleed between wavelength channels, a sleeve was created via removal of the clear plastic base of a black 96-well plate (Corning, Sigma-Aldrich, MO, USA). A benchtop power supply (Iso-Tech, IPS-603, UK) was used to power the array. Array design is depicted in Supplementary Fig. 1 and described in detail by Hadis et al. [42].

### LED array characterisation

#### Spectral characterisation

A UV–Vis spectrometer (USB400, Ocean Optics, UK) coupled to a 200-μm optical fibre and opaline glass CC3 cosine corrector was used to assess emitted spectral irradiance and wavelength values (calibrated to a National Institute of Standards and Technology (NIST) traceable light source (Micropack DH200/Ocean Optics, UK)). Spectral irradiance measurements from each individual LED were recorded utilising OceanView software (Ocean Optics, UK), providing an average emitted spectral irradiance of ~ 24mW/cm^2^ (Table 1). Acquisition of these measurement facilitated calculation of radiant exposure (J/cm^2^, energy received per unit area) through multiplication of irradiance output (W/cm^2^) by irradiation period (Table 1). Detailed experimental procedure and additional characterisation measures are reported by Hadis et al. [42].

**Table 1:** Details of parameters emitted from our 96-well LED array at each wavelength channel (range: 400–830 nm). Irradiance (mW/cm^2^) and recorded wavelength (nm) were measured using a UV–Vis spectrometer and beam area (cm^2^) was determined using beam profilometry, in which a 96-well black clear bottom plate was placed above the LED array during measurements to replicate experimental conditions (n = 6 for each wavelength channel). Radiant exposure is also reported for each time interval (30–480 s) and was calculated through multiplication of average irradiance output (W/cm^2^) with irradiation time (s). Experimental design and setup are described in detail in Serrage et al. [43]. All parameters were determined over a 240-s irradiation period. LED array design and experimental setup is provided in Supplementary Fig. 1

Manufacturer reported wavelength (nm)	Measured wavelength (nm)	Irradiance (mW/cm^2^)	Beam area (cm^2^)	Radiant exposure (J/cm^2^)				
				30 s	60 s	120 s	240 s	480 s
400	399.68 ± 1.02	24.14 ± 1.42	0.32 ± 0.04	0.72	1.45	2.9	5.79	11.59
450	446.85 ± 2.27	25.16 ± 1.97	0.24 ± 0.02	0.75	1.51	3.02	6.04	12.09
525	523.83 ± 1.23	23.52 ± 2.81	0.26 ± 0.02	0.71	1.41	2.82	5.64	11.29
660	662.24 ± 0.6	24.56 ± 1.40	0.32 ± 0.04	0.74	1.47	2.95	5.89	11.79
740	735.51 ± 1.08	22.90 ± 1.46	0.28 ± 0.04	0.69	1.37	2.75	5.5	10.99
810	817.87 ± 0.78	22.93 ± 2.35	0.27 ± 0.02	0.69	1.38	2.75	5.5	11.01
830	830.03 ± 2.87	24.29 ± 3.26	0.25 ± 0.01	0.73	1.49	2.92	5.83	11.66
White	455.73 ± 0.33	26.57 ± 2.53	0.25 ± 0.01	0.8	1.59	3.19	6.38	12.75
Average		24.25 ± 1.22	0.24 ± 0.02	0.73 ± 0.04	1.46 ± 0.07	2.91 ± 0.15	5.82 ± 0.29	11.65 ± 0.58

#### Beam profile

A charge-coupled device (CCD) camera beam profiler (SP620, Ophir, Spiricon, Jerusalem, Israel) was employed to measure spatial distribution of irradiance. Wavelengths between 400 and 830 nm were measured through a target screen (N-BK7 ground glass diffuser, Thorlabs, NJ, USA) to ensure reliable indication of the spatial distribution of irradiance at the plane of the adherent cells. A 50-mm closed-circuit television lens (CCTV; Ophir, Spiricon) attached to the camera and focused on the base of each well. Following linear, optical and ambient light correction, images were recorded using BeamGage software. Detailed experimental procedures have been previously reported [42]. Calculated beam area is described in Table 1 and representative beam profile images have been previously reported by Serrage et al. [43].

### Biological responses

#### Bacterial culture

Bacterial stocks of *Porphyromonas gingivalis* (*P. gingivalis,* ATCC 33,277) and *Fusobacterium nucleatum* sp. *Polymorphum* (*F. nucleatum,* ATCC 10,953) were acquired from the American Type Culture Collection (ATCC, USA) and routinely cultured in 3.7% w/v Sabouraud Dextrose Broth (OXOID) for < 10 days under anaerobic conditions (37 °C). Following initial culture, suspensions were centrifuged 3 × (Durafuge 100, Precision, Expotech, USA), resuspended in PBS to a working concentration of 1 × 10^8^ CFU/ml and heat-inactivated at 80 °C for 1 h. Viability of heat-inactivated cultures was assessed on fastidious anaerobe agar (FAA, OXOID). Purity of cultures was also assessed using Gram stain [44] and species-specific polymerase chain reaction (PCR) undergone as previously reported by Takeshita et al. [45]. Suspensions were stored at − 20 °C until required for experimentation.

#### Primary human gingival fibroblast (GF) culture and exposure

GFs were obtained from waste gingival tissue harvested during surgical extraction of impacted third molars in healthy adult subjects and grown from explants (approved by University of Birmingham Ethics Committee, RG_12-020) and isolated as described previously [46]. Details regarding age, sex and ethnicity of gingival fibroblast donors were not disclosed during this study. GF morphology was assessed by light microscopy, in which a characteristic spindle-shaped morphology was observed [47]. Relative expression of vimentin was also evaluated via real-time PCR as described by Liu et al. [46].

GFs (pooled from three individuals, all at passages 5–8) were cultured in monolayers in Dulbecco’s modified eagle medium without phenol red (DMEM, Gibco, UK) supplemented with 10% v/v foetal bovine serum (FBS), 1% v/v penicillin/streptomycin (P/S) and 1% v/v L-glutamine (Sigma-Aldrich, UK). Fibroblasts were seeded into 96-well black clear bottom plates (7000 cells/well) and incubated overnight (37 °C, 5% CO2). Cultures were then washed 3 × with PBS and resuspended in DMEM ± *Escherichia coli* LPS (026:B6 1–20 μg/ml, Sigma-Aldrich, UK), heat-inactivated *F. nucleatum* (10–500:1 multiplicity of infection, MOI) or heat-inactivated *P. gingivalis* (10–500:1 MOI). Concentrations of periodontopathogens applied to GFs were selected to mimic those reported in periodontal pockets [48, 49].

#### Irradiation of cultures

At 16 h following seeding, cell culture plates were removed from the incubator and placed upon a bespoke 96-well LED array [41], in which LEDs were concentrically aligned with wells from above, and the distance between LEDs and the monolayer of adherent cells was fixed at 3 mm. Cultures were irradiated from directly beneath using the LED array for time intervals up to 480 s. Irradiation parameters, including irradiance, beam area and radiant exposure for each time interval at each wavelength are provided in Table 1. The effects of irradiation on media temperature are provided in Supplementary Fig. 2, in which no significant difference between wavelength channels was observed. For high-throughput assessment of parameters on cell metabolic activity, forty-eight culture wells were irradiated simultaneously with one column of LEDs used as non-irradiated controls (n = 6, Supplementary Fig. 1). Following the selection of parameters of interest, eighteen culture wells were irradiated simultaneously, and one column of LEDs was used as non-irradiated controls (n = 6). For studies examining the effects of PBM following application of a pro-inflammatory stimulus, one column of LEDs was used as the non-irradiated unstimulated control (n = 6) and a second column as the non-irradiated bacterially stimulated control (n = 6). All experiments were performed in triplicate. Following irradiation, cultures were incubated until used for further analysis.

### Cell metabolic activity assay

3-(4,5-dimethylthiazol-2-yl)-2,5-diphenyltetrazolium bromide (MTT) assay (Sigma-Aldrich, UK) was utilised to assess changes in cell metabolic activity. MTT is converted to insoluble formazan via mitochondrial enzymes, the formation of which does not correlate with changes in cell proliferation [50, 51]. The assay was performed as described by Serrage et al. [43].

### Adenosine triphosphate (ATP) assay

A luminescent ATP detection assay was utilised to detect total levels of cellular ATP 24 h post-irradiation according to the manufacturer’s instructions (Luminescent ATP detection assay, Abcam, UK).

### Reactive oxygen species (ROS) assay

ROS formation was assessed using 2′, 7′-dichlorodihydrofluorescein diacetate (H2DCFDA) fluorescent probe (Thermo-Fischer Scientific, UK). Free radicals catalyse the conversion of H2DCFDA to its fluorescent bi-marker DCF, enabling quantification of ROS production. At 24 h post-irradiation media was aspirated, cells were washed with PBS and treated with 10 μM H2DCFDA and incubated for 1 h at 37 °C [52]. Fluorescence was read using a fluorimeter (Twinkle LB 970, Berthold Industries Ltd, Germany, 485 nm/535 nm, excitation/emission respectively).

### Enzyme-linked immunosorbent assay (ELISA*)*

GF supernatants were assayed for human total interleukin-8 (IL-8) and transforming growth factor-β1 (TGFβ1) by ELISA according to the manufacturer’s protocol (Human IL-8/TGFβ1 ELISA, both R and D systems, Biotechne, MN, USA).

### Reverse transcription–polymerase chain reaction (RT-PCR*)*

Following the initial culture of GFs, cells were lysed, and RNA extracted from lysates according to the manufacturer’s instructions (RNeasy mini kit, Qiagen, UK). Reverse Transcription (RT) of RNA samples was performed to produce high-quality cDNA samples using a Bioline Tetro cDNA kit (Bioline, UK) according to the manufacturer’s guidelines. A primer mix was prepared containing up to 2 μl cDNA, 2 μl primer (50 pmol), 8.5 μl RNase free water (Qiagen, UK) and 12.5 μl Biomix red (Bioline, UK). The following primers were employed for this study: transforming growth factor-β receptor 1 (TGFβR1: Forward: 5’-CGTTACAGTGTTTCTGCCACCT-3’, Reverse: 5’-AGACGAAGCACACTGGTCCAGC-3’) and Interleukin-8 (IL-8 F: 5’- CGCCTTAGCGCCCACTGCTCCTGT-3’, R: 5’- GGGGCGGGACCTCAGCTGCAC-3’). These genes were selected to explore ROS-induced changes in IL-8 and TGFβ signalling. Conditions for PCR were as follows: 94 °C for 5 min, 30 cycles of 60 °C, 72 °C and 94 °C for 1 min each and an extension step at 72 °C for 10 min. Relative quantities of cDNA were normalised against the housekeeping gene glyceraldehyde-3-phosphate dehydrogenase (GAPDH). PCR products were confirmed using SYBR red (0.01% v/v, Invitrogen, UK) containing agarose gel electrophoresis (1.5% w/w agarose). Images were captured under Ultraviolet trans-illumination utilising Genesnap software on the G:box imaging system (Syngene, UK). Semi-quantification of PCR bands was undergone using GeneTools (Syngene, UK).

### Statistical analysis

All experiments were performed in triplicate, with *n* = 6 replicates per treatment group per experiment. Data was processed utilising Excel software (Microsoft) and analysis performed using SigmaPlot software (Systat Software Inc, UK). All data was analysed using a general linear model (GLM) followed by a one-way ANOVA test followed by a Tukey test to determine significant differences between non-irradiated controls (bacterially stimulated or otherwise) and light irradiation–treated groups (*p* < 0.05).

## Results

### The effects of PBM are wavelength- and dose-dependent

**Fig. 1:**
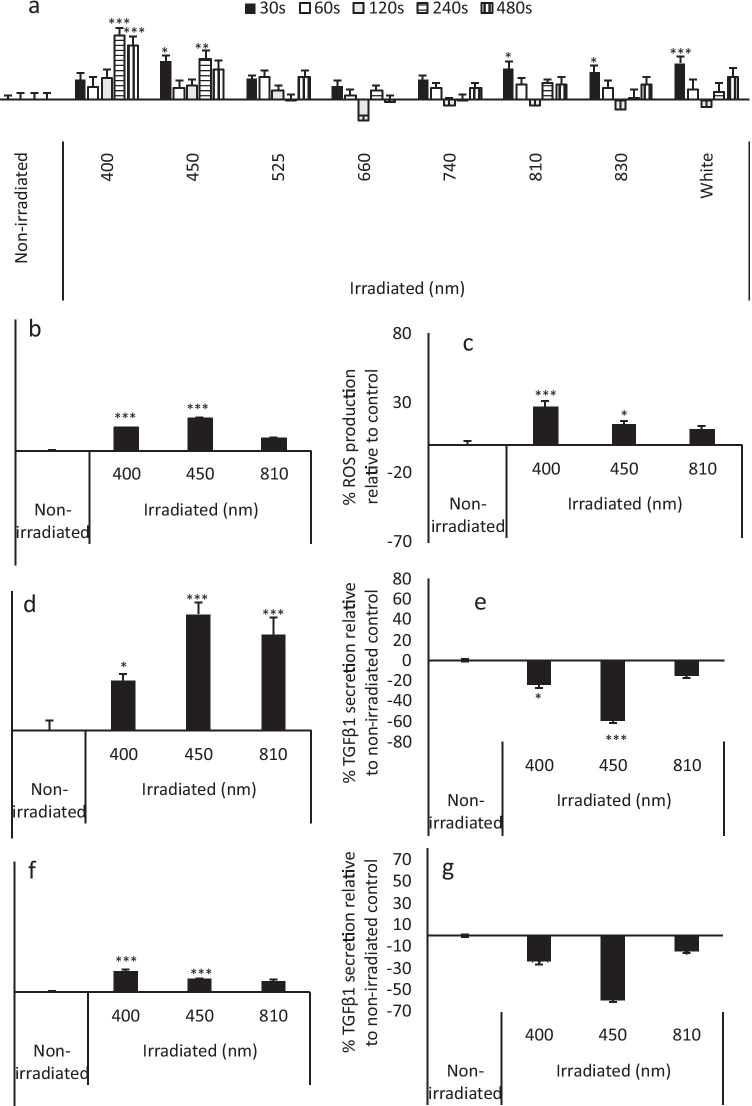
Blue light modulates markers for mitochondrial activity and inflammation. **a** High-throughput analysis of various wavelengths (400–830 nm) and irradiation periods (30–240 s) on cell metabolic activity of pooled primary human gingival fibroblasts (B19, B16, B15 samples, 24mW/cm^2^, 0.72–5.76 J/cm^2^, 30–240 s) 24 h post-irradiation was assessed via MTT assay. The effects of PBM (400 nm, 450 nm or 810 nm, 5.76 J/cm^2^, 24mW/cm^2^) on markers for mitochondrial activity and inflammation were measured 24 h post-irradiation. PBM-induced changes in mitochondrial activity were assessed via ROS (**b**) and ATP (**c**) assays. Cell lysates were collected, RNA extracted and RT-PCR employed to elucidate PBM-induced changes in IL-8 (**d**) and TGFβR1 (**e**) gene expression. Evaluation of PBM on IL-8 (**f**) and TGFβ1 (**g**) secretion was undergone via collection of supernatants and analysis by ELISA. All experiments were performed in triplicate and presented as mean ± SD. Significance was assessed using one-way ANOVA followed by Tukey test and is indicated by ***p < 0.001, **p < 0.01 and *p < 0.05 relative to the non-irradiated control, where all data is shown as a percentage of the non-irradiated control, where the non-irradiated control was normalised to 0%

The biphasic effects of PBM (400–830 nm, 0.72–11.52 J/cm^2^) on non-stimulated GFs using MTT as a marker for mitochondrial activity were evaluated. High-throughput screening of parameters revealed 400 nm or 450 nm light at 5.76 J/cm^2^ (240 s) induced 14.6% and 9.2% increases in mitochondrial activity relative to the non-irradiated control (*p* < 0.01, Fig. 1a) and thus these parameters were selected for further evaluation. A wavelength of 810 nm and irradiation period of 240 s were also selected for further study. Whilst no significant increase in MTT was detected at 810 nm, it is frequently used in vitro and therefore findings could be compared with current literature [53, 54].

### Blue light modulates markers for mitochondrial activity and inflammation

The effects of PBM are hypothesised to be associated with mitochondrially induced temporal changes in ATP and ROS production [30, 55]. Exposure to 400 nm and 450 nm light induced significant increases in both ROS and ATP production relative to the untreated control (*p* < 0.05, Fig. 1b–c).

ROS-induced changes in IL-8 and TGFβ signalling were also assessed. Irradiation at 450 nm (5.76 J/cm^2^) induced 75% increases in both IL-8 gene expression (*p* < 0.001, Fig. 1d) relative to the untreated control. Comparatively, 450 nm light decreased TGFβ-R1 gene expression (*p* < 0.001, Fig. 1e, Supplementary Fig. 3).

### Effects of bacterial stimuli on markers for inflammation are dose-dependent

**Fig. 2:**
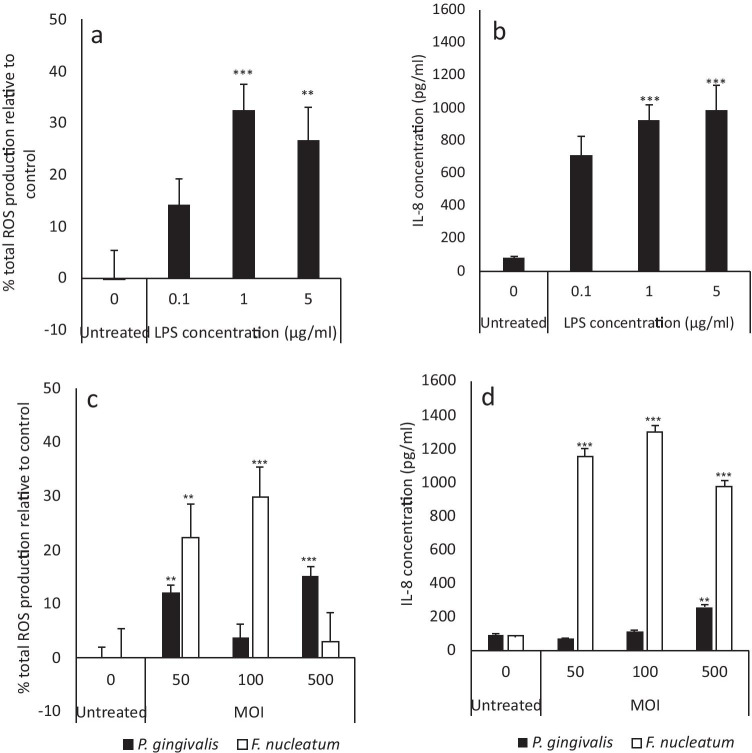
LPS, *F. nucleatum* and *P. gingivalis* modulate markers for inflammation in a dose-dependent manner. Following 24 h incubation, pHGFs (B15, B16 and B19, p5-8) ± LPS (1-5 μg/ml)/heat-inactivated *F. nucleatum* (50–500: 1 MOI)/heat-inactivated *P. gingivalis* (50–500:1 MOI) were assayed for ROS production (ROS assay **a**, **c**) and IL-8 secretion (ELISA, **b**, **d**). All experiments were performed in triplicate and presented as mean ± SD. Significance was assessed using one-way ANOVA followed by the Tukey test and is indicated by ***p < 0.001, **p < 0.01 and *p < 0.05 relative to the non-irradiated control, where all data is shown as a percentage of the corresponding untreated control, where the untreated control was normalised to 0%

The minimum dose of bacterial stimuli to induce increases in markers for inflammation (ROS and IL-8) was assessed to mimic the chronic inflammatory phenotype observed in periodontitis [56]. LPS, heat-inactivated *P. gingivalis* or *F. nucleatum* elicited their effects on ROS production and IL-8 secretion in a dose-dependent manner (Fig. 2). A concentration of 1 μg/ml of LPS, a MOI of 100:1 *F. nucleatum* and a MOI of 500:1 *P. gingivalis* were thus selected for experimental application. *P. gingivalis* induced significantly lower levels of IL-8 secretion relative to LPS- and *F. nucleatum*–treated GFs (*p* < 0.001). Indeed, comparable concentrations have also been used in the literature, when assessing the effects of bacterial stimuli on inflammation from oral cell types [48, 49, 57].

### Bacterial stimulus–induced changes in mitochondrial activity and inflammation are modulated by blue light

**Fig. 3:**
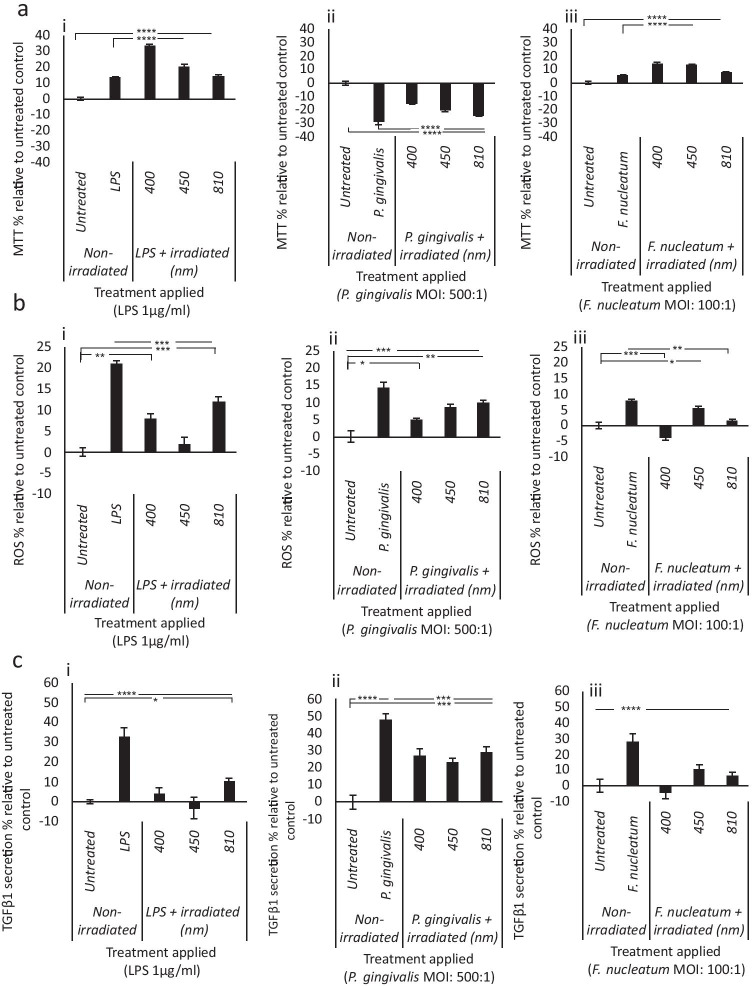
PBM modulates bacterially induced changes in markers for cell metabolic activity and inflammation. ppHGFs (B15, B16 and B19, p5-8) ± LPS (1 μg/ml, i)/heat-inactivated *P. gingivalis* (500:1 MOI, ii)/heat-inactivated *F. nucleatum* (100:1 MOI, iii) were subsequently treated with PBM (400–810 nm, 5.76 J/cm^2^, 24mW/cm^2^). PBM-induced changes in bacterially stimulated changes in **a** cell metabolic activity (MTT assay), **b** ROS production (marker for mitochondrial activity, ROS assay) and **c** TGFβ1 secretion (marker for inflammation, ELISA on collected supernatants) were then assessed 24 h post-irradiation. All experiments were performed in triplicate and presented as mean ± SD. Significance was assessed using one-way ANOVA followed by Tukey’s test and results are indicated by ****p < 0.0001, ***p < 0.001, **p < 0.01 and *p < 0.05 relative to the respective non-irradiated controls. All data is shown as a percentage of the non-irradiated control, where the non-irradiated control in each experimental group (LPS, *F. nucleatum* or *P. gingivalis*) was normalised to 0%

Bacterially stimulated cultures were assessed for the effects of PBM on markers for cell metabolic and mitochondrial activity (MTT, ROS) and inflammatory response (TGFβ1, IL-8). Cultures treated with LPS or *F. nucleatum* exhibited a significant increase in MTT (*p *< 0.0001, Fig. 3a i and iii). Interestingly, blue light (400–450 nm, 5.76 J/cm^2^) further increased levels significantly higher than the respective bacterially treated control (*p *< 0.0001, Fig. 3a i and iii). *P. gingivalis*–induced decreases (− 29%) in MTT were alleviated via application of blue light (400–450 nm, *p* < 0.0001, Fig. 3a ii). Cell counts were also performed, and data indicated that irradiation of bacterially stimulated cultures induced no significant effect on cell numbers relative to the respective non-irradiated controls (Supplementary Fig. 4). These data indicated that the irradiation conditions applied had no significant effect on cell growth.

Bacterially stimulated cultures (LPS/*F. nucleatum/P. gingivalis*) treated with 400 nm light exhibited significant decreases in ROS production relative to their respective bacterially stimulated controls (*p* < 0.01 LPS, *p* < 0.001 *F. nucleatum* and *P. gingivalis* Fig. 3b i–iii), to levels comparable with the untreated control. Blue light (400 nm) also restored TGFβ1 secretion to levels comparable to the untreated control for both LPS- and *F. nucleatum–*treated cultures (Fig. 3c i and iii, *p* < 0.0001).

**Fig. 4:**
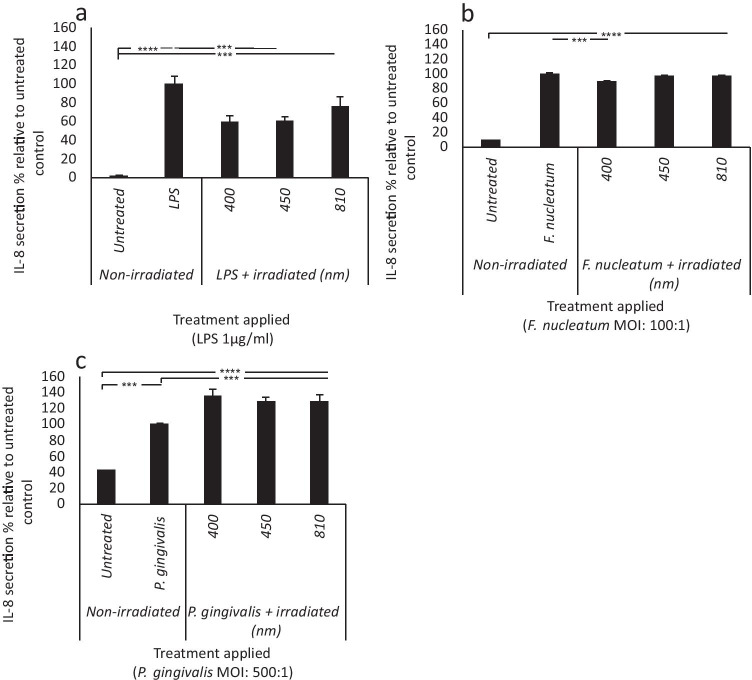
The effects of PBM on IL-8 secretion are bacterial stimulus–dependent. pHGFs (B15, B16 and B19, p5-8) ± LPS (1 μg/ml, **a**)/heat-inactivated *F. nucleatum* (100:1 MOI, **b**)/heat-inactivated *P. gingivalis* (500:1 MOI, **c**) were subsequently treated with ± (400–810 nm, 5.76 J/cm^2^, 24mW/cm^2^). PBM-induced changes in bacterially stimulated changes in IL-8 secretion were then assessed 24 h post-irradiation. All experiments were performed in triplicate and presented as mean ± SD. Significance was assessed using one-way ANOVA followed by Tukey’s test and is indicated by ****p < 0.0001 ***p < 0.001, **p < 0.01 and *p < 0.05 relative to the respective non-irradiated controls. All data is shown as a percentage of the corresponding stimulated control, where the stimulated control in each experimental group (LPS, *F. nucleatum* or *P. gingivalis*) was normalised to 100%

Analysis of IL-8 secretion by ELISA revealed 400 nm light application was able to induce significant reductions in IL-8 secretion from LPS (− 40%) and *F. nucleatum* (− 10%) stimulated cultures relative to their respective treated controls (Fig. 4a–b, *p* < 0.001). Application of 400 nm light induced 36% increases in IL-8 secretion relative to the *P. gingivalis* stimulated control respectively (Fig. 4c, *p* < 0.001).

## Discussion

This report provides novel evidence for the biomodulatory effects of blue light on GFs. We demonstrate the use of a high-throughput system for selection of PBM treatment parameters required to induce a response from GFs. In which, a range of parameters were screened simultaneously utilising a novel 96-well LED array. Where, blue light (400–450 nm) proved most effective in modulating both markers for mitochondrial activity and inflammation.

Blue light is not commonly used in PBM studies due to its proximity to the ‘damaging’ ultraviolet (UV) band of the electromagnetic spectrum. However, it has been reported that blue light at doses < 55 J/cm^2^ can induce beneficial effects including reduced inflammation and improved healing times following oral surgery [30]. Indicating, blue light could prove an effective treatment modality within a narrow therapeutic range.

The direct application of low-dose blue light on GFs has been assessed by relatively few authors, with PBM at > 100 J/cm^2^ being required to induce increased mitochondrial ROS production, accelerated oxidative stress and inhibited cell proliferation [21, 22, 58–60]. Comparatively, our report now provides new evidence for the biomodulatory effects of low-dose blue light on GFs at < 10 J/cm^2^. Where, parameters employed exert no cytotoxic effects on cell cultures.

Previous reports indicate blue light could excite flavin (complexes I–II) and porphyrin (complex IV) containing complexes of the ETC, inducing increases in mitochondrial activity and its associated secondary messengers, ROS and ATP [30, 61]. The 400 nm light induced significant increases in markers for mitochondrial activity (MTT Fig. 1a, ATP and ROS, Fig. 1b–c), which corroborates with findings previously published by our group, wherein blue light modulated the real-time mitochondrial activity of muscle-derived cell types at doses < 10 J/cm^2^ [43].

Blue light also exhibited the ability to modulate downstream TGFβ signalling by reducing TGFβ1 secretion and TGFβR1 gene expression (Fig. 1g and e,* p* < 0.001). The efficacy of blue light inhibiting TGFβ-induced cell differentiation has been previously reported [62]. Differentiated fibroblasts known as myofibroblasts are uncommon in healthy gingival tissue and thus blue light may regulate TGFβ-induced fibroblast differentiation by modulating TGFβ1 secretion and promoting the autocrine regulation of TGFβR1 gene expression [63].

It was also observed that NIR PBM (810 nm, 5.76 J/cm^2^, *p* < 0.05) induced significant decreases in TGFβ1 secretion. Comparatively, literature surrounding the effects of red and NIR PBM on TGFβ signalling remains conflicting [64–67]. In their review, Mokoena et al. [68] reported authors observed both decreases and increases in TGFβ signalling following red and NIR PBM. Indicative of the parameter dependent response to PBM and further solidifying the requirement for the correct reporting and recording of treatment parameters.

The effects of PBM on periodontal pathogen–stimulated oral fibroblasts were also evaluated. Current literature generally studies the effects of red and NIR PBM on LPS-stimulated gingival fibroblasts [18, 24, 25, 28, 36]. Thus, alongside LPS, two species critical in oral biofilm formation and induction of periodontal disease were selected to assess the bacterial stimulus–dependent response to PBM, *F. nucleatum* and *P. gingivalis.*

LPS (1 μg/ml), *F. nucleatum* (100:1 MOI) and *P. gingivalis* (500:1 MOI) induced significant increases in both ROS and IL-8 relative to the respective untreated control (*p* < 0.001, Fig. 2). However, the levels of IL-8 secretion induced by *P. gingivalis* were 5- and 3.6-fold lower than those observed for *F. nucleatum* and LPS, which may provide evidence of the possible differing contributions of oral bacterial species to inflammation in vivo*.*

Changes in mitochondrial function have been shown to influence the rate of formazan formation and thus MTT assay output [69]. LPS and *F. nucleatum* induced significant increases in MTT relative to the untreated control (Fig. 3a, *p* < 0.05). As LPS has been reported to induce mitochondrial biogenesis [70], it is hypothesised that LPS and *F. nucleatum* induced increases in mitochondrial function, corroborated by their ability to induce ROS production (Fig. 3b, *p* < 0.05). Comparatively, *P. gingivalis* decreased cell metabolic activity possibly due to perturbed mitochondrial function [71]. However, application of blue light (400–450 nm, 5.76 J/cm^2^) induced increased cell metabolic activity of LPS, *F. nucleatum*– and *P. gingivalis*–treated cultures (Fig. 3a). This indicated a possible PBM-dependent increase in mitochondrial activity and not cell proliferation, where bacterial stimulation with or without light application had no significant effect on cell number relative to the control (Supplementary Fig. 4).

The effects of PBM on bacterial stimulus–induced changes in ROS production were also evaluated, where the primary source of endogenous ROS is the mitochondria [72]. The 400 nm light induced significant decreases in ROS production relative to the respective bacterially stimulated controls (Fig. 3b), which may be indicative of a possible feedback loop induced by PBM, leading to the upregulation of antioxidant regulation proteins such as nuclear factor erythroid 2–related factor 2 (Nrf2) [30].

PBM (400 nm) also induced significant decreases in bacterially stimulated TGFβ1 secretion (Fig. 3c). Following damage to gingival tissue during periodontitis, TGFβ induced myofibroblast formation can occur, which can lead to delayed wound healing and fibrosis of affected tissue [62]. Thus, blue light could be an effective modality in modulating bacterially stimulated TGFβ signalling in periodontitis.

Concomitantly, 400 nm light also induced significant reductions in LPS and *F. nucleatum* stimulated increases in IL-8 secretion (Fig. 4) indicating the possible efficacy of blue light in modulating oral inflammation. Indeed, the effects of NIR light in modulating LPS-induced changes in IL-8 secretion have been reported [36]. However, our results do not entirely corroborate these findings. We found that NIR light (810 nm) had no significant effect on LPS-induced changes in IL-8 secretion. This outcome is possibly due to the different radiant exposure used in this study (5.76 J/cm) compared with previous studies which reported modulation of LPS-induced markers for inflammation at 0.5–3 J/cm^2^ [19, 36].

In comparison, blue and NIR light exacerbated *P. gingivalis*–induced increases in IL-8 secretion. However, levels of IL-8 secretion induced by *P. gingivalis* were significantly lower than those induced by LPS or *F. nucleatum* (Fig. 2). Cytokines such as IL-8 play a critical role in the maintenance of cellular homeostasis and thus are commonplace in healthy gingival tissue [73]. PBM is documented to induce small increases in markers for inflammation from ‘healthy tissue’ but induces decreases in chronically inflamed tissue [30]. We hypothesise that application of *P. gingivalis* does not induce IL-8 secretion to levels reaching a chronic inflammatory threshold, thus blue light induces further increases, possibly alerting the immune system of the presence of a bacterial stimulus and ensuring effective management of infection.

## Conclusion

We demonstrate novel in vitro evidence of the biomodulatory effects of low-dose blue light. In a model for ‘oral health’, we observed that blue light (400–450 nm, 5.76 J/cm^2^) induces small increases in inflammatory markers which could prove beneficial in the maintenance of cellular homeostasis and thus oral health. Comparatively, blue light (400–450 nm, 5.76 J/cm^2^) induces decreases in IL-8 and ROS from periodontally stimulated GFs, which could prove effective in managing the excessive inflammatory response observed in periodontitis. These findings provide novel evidence of clinically translatable irradiation parameters for the modulation of inflammation associated with oral disease.

## Supplementary Information

Below is the link to the electronic supplementary material.

Supplementary file1 (DOCX 14602 KB)

## Data Availability

Data available within the article or its supplementary materials.
