# Supplementary material for: Photobiomodulation of oral fibroblasts stimulated with periodontal pathogens
Source: Lasers Med Sci. 2021 May 15;36(9):1957–69. doi: 10.1007/s10103-021-03331-z (PMC8593050; doi:10.1007/s10103-021-03331-z)

**Methods**

*Cell confluency assay*

To assess cell confluency a Tecan Spark® (Tecan Group Ltd, Switzerland) multimode microplate reader was employed. A standard curve was established at 3000-10000 cells/well to enable correlation of confluency with cell number (n=3). Plates were then incubated overnight (37ᵒC, 5% CO₂). Cultures were then treated as described in section 1.2 and incubated for a further 8-120 h. Levels of cell confluence were then assessed through selection of a pre-set algorithm to measure cell confluence on the Spark® dashboard. Once levels of confluence were assessed, the wells used to produce a standard curve were washed with PBS, treated with Trypsin ethylenediaminetetraacetic acid (Sigma Aldrich, UK, 20µl/well) for 5 minutes (37ᵒC, 5% CO₂) and mixed with an equal volume of DMEM containing 10% FCS. Cells were counted using Tecan Spark® counting chips as per the manufacturer’s instructions. These data were then used to correlate confluency with cell number.

*Supplementary figures*


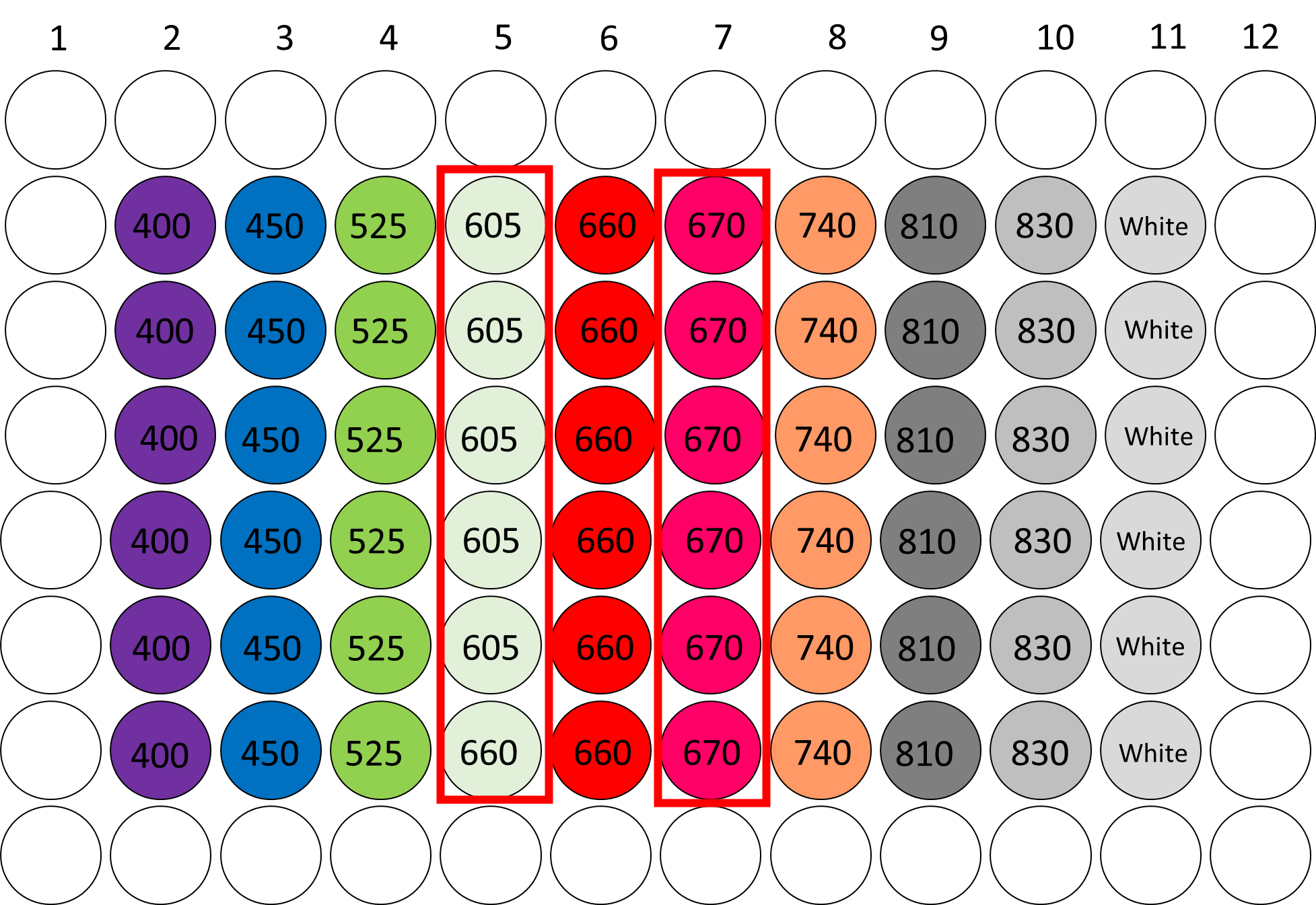

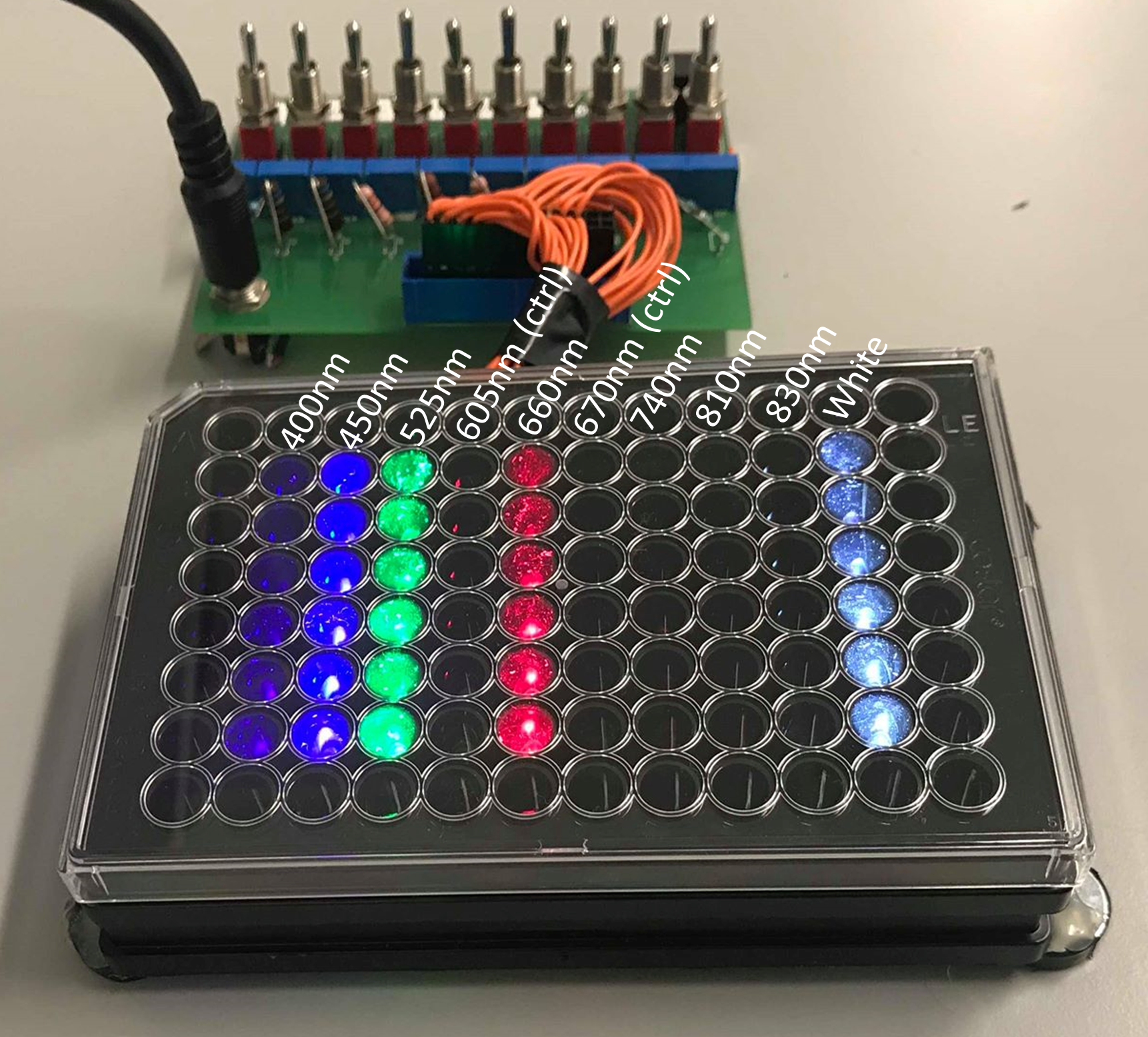


a

b

**Figure 1: LED array layout and experimental setup**. a) Wavelengths (nm) used across the array, channels not used during experimentation are highlighted by red boxing and were used as non-irradiated controls during experimental application. b) Experimental setup for the LED array utilised a black clear bottom 96-well plate placed upon the array to enable uniform irradiation of cultures from the base of the well, all wavelength channels are highlighted for reference. Switches are highlighted with the red box, where individual wavelength channels could be controlled.


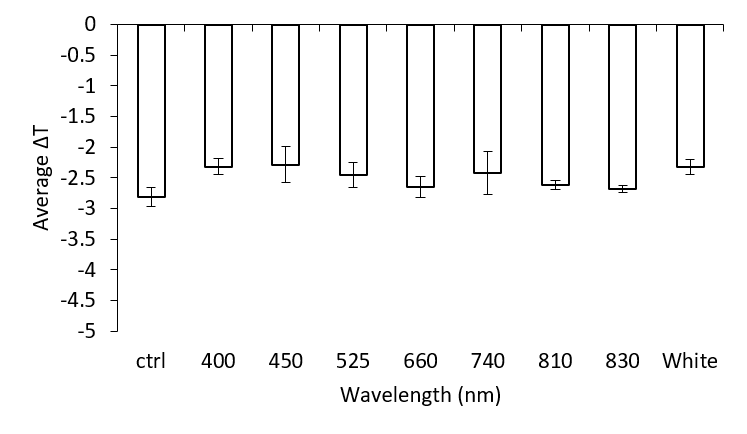


**Figure 2: Irradiation does not affect media temperature.** DMEM phenol red free media was aliquoted into a 96 well black clear bottom plate, warmed to 37⁰C and irradiated for 480s (400 – 830 nm, 24mW/cm², 11.72J/cm²). Changes in temperature during irradiation were assessed in real time via insertion of a thermistor into wells (n=6 per wavelength channel) and recorded using PicoLog software (Pico Technology, Cambridgeshire, UK). Experimental design and setup is described in detail by Serrage et al. Significant differences in average temperature change between wavelength channels was assessed via one way ANOVA followed by Tukey test.


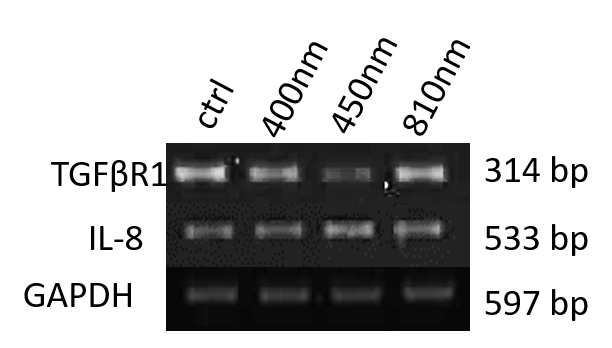


**Figure 3:** Representative PCR images of TGFβR1, IL-8 and GAPDH (control) expression from pool pHGFs (B15, B16, B19, p5-8) ± 400-810 nm (5.76 J/cm², 24 mW/cm²) irradiation. Effects on gene expression were evaluated 24 h post – irradiation and all experiments were performed in triplicate. All gene expression was normalized against GAPDH expression.

**Figure 4: PBM induced no significant effect on cell proliferation of bacterially-stimulated pHGF cultures.** pHGFs were seeded at 7000 cells/well (pool, p5-8, n=3 technical replicates), incubated overnight and stimulated with E. coli LPS (1µg/ml)/heat-inactivated F. nucleatum (100:1 MOI)/ P. gingivalis (500:1 MOI) and subsequently treated with PBM (400 – 810 nm, 24mW/cm², 5.76 J/cm²) and incubated for a further 120 h. Changes in cell number were assessed at 8 – 120 h post-irradiation and analyzed using the Tecan Spark® platform. In which, cell confluency was assessed and correlated to cell number via automated cell counts of control wells. % changes in cell number relative to the respective untreated control at each time point were then calculated for PBM ± (A) LPS, (B) F. nucleatum and (C) P. gingivalis treated cultures. All experiments were performed in triplicate and are presented as mean ± SD. Significance was measured using one-way ANOVA followed by Tukey test where means not sharing the same letter are significantly different (p<0.05).


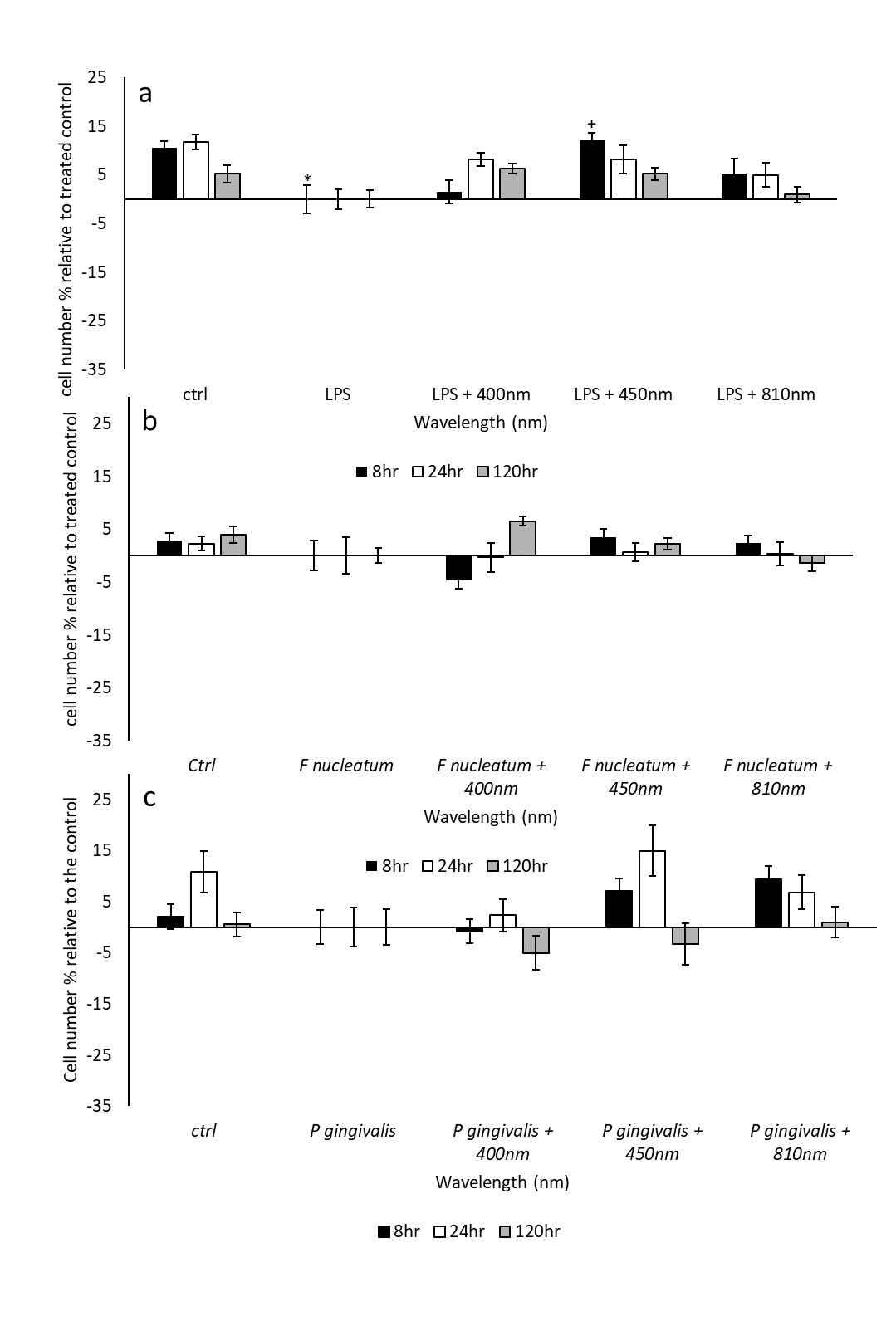


**Figure 5:** **The effects of PBM on cell number, 8, 24 and 120h post-irradiation (24mW/cm², 240s, 5.76J/cm²).** pHGFs were seeded at 7000 cells/well (pool, p5-8, n=3 technical replicates), incubated overnight and subsequently irradiated the following morning. Changes in cell number were then analyzed using the Tecan Spark® platform. (A) Cell growth curves at 8, 24 and 120 h post-irradiation and (B) % changes in cell number relative to the respective untreated control at each time point. Significance was measured using one-way ANOVA followed by Tukey test where means not sharing the same letter are significantly different (p<0.05).


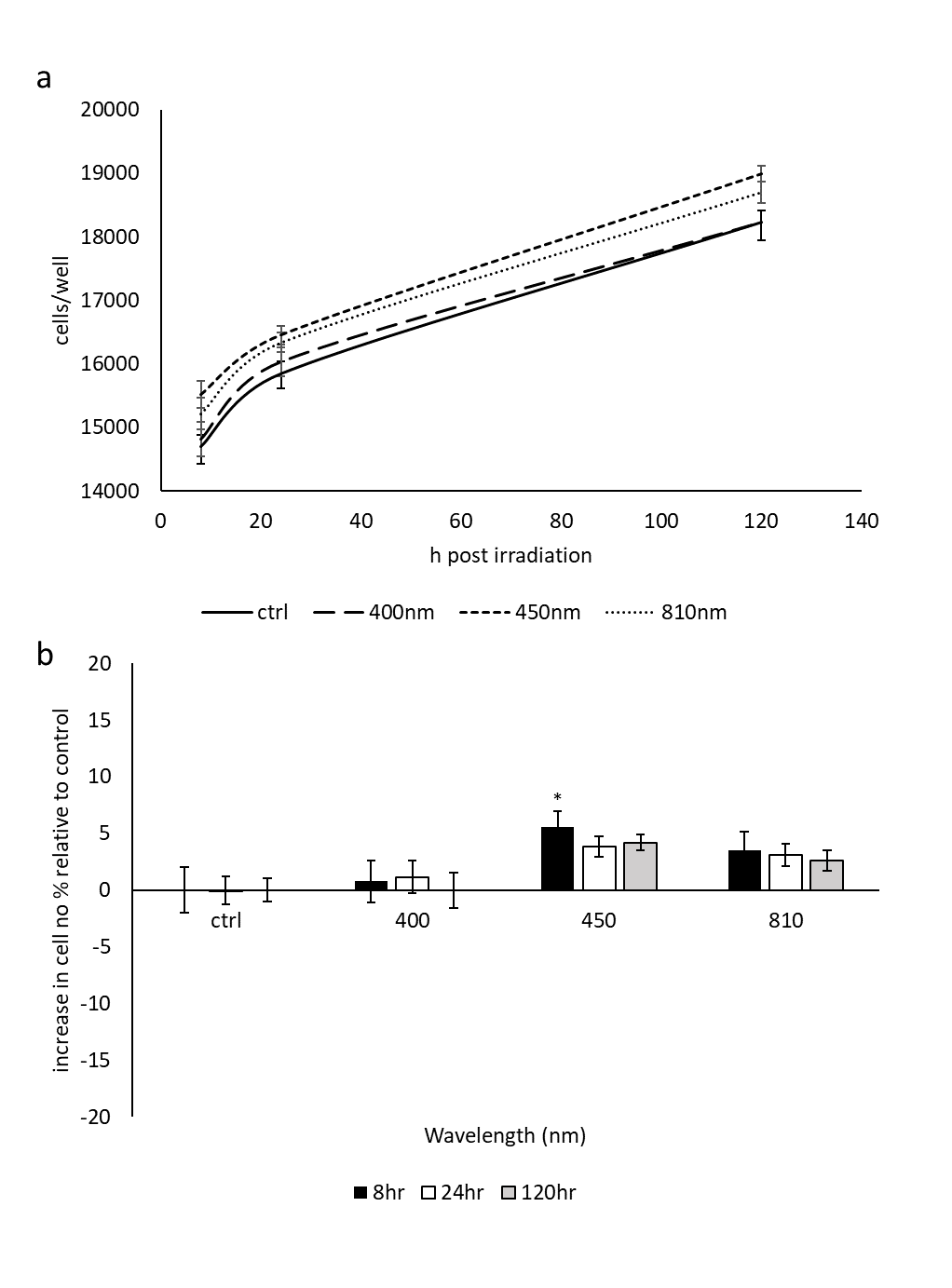

Supplement: Supplementary file 1 — Supplementary file1 (DOCX 14602 KB) [file 10103_2021_3331_MOESM1_ESM.docx]
